# Photokinetics of Dacarbazine and Nifedipine under polychromatic light irradiation and their application as new reliable actinometers for the ultraviolet range

**DOI:** 10.1038/s41598-022-11570-5

**Published:** 2022-05-10

**Authors:** Mounir Maafi, Mohammed Ahmed Al-Qarni

**Affiliations:** 1 Leicester School of Pharmacy, De Montfort University, The Gateway, Leicester, LE1 9BH UK; 2 Department of Pharmaceutical Chemistry, College of Pharmacy, Taif University, P.O. Box 11099, Taif, 21944 Saudi Arabia

**Keywords:** Medical research, Chemistry, Energy science and technology, Materials science

## Abstract

The photokinetic behaviour of drugs driven by polychromatic light is an area of pharmaceutics that has not received a lot of attention. Most often, such photokinetic data is treated by thermal kinetic models (i.e., the classical 0th-, 1st- or 2nd-order equations). Such models were not analytically derived from the rate-laws of the photodegradation reactions. Polychromatic light kinetic modelling is hence of importance, as a means to providing adequate toolkits and metrics. This paper aims at proposing two reliable drug-actinometers useful for polychromatic UVA range. The general actinometric methodology offered here is also useful for any drugs/materials obeying a primary photoprocess where both reactant and photoproduct absorb the incident light, of the $AB{(1\Phi )}_{{\varepsilon }_{B}\ne 0}$ type. The present method has been consolidated by the η-order kinetics. This framework further demonstrated the lamp-specificity of actinometers. Overall, Dacarbazine and Nifedipine photodegradations obeyed η-order kinetics, and stand as effective actinometers that can be recommended for the ICH Q1b photostability testing.

## Introduction

Photostability of drugs is an important part of drugs’ stability studies^1–3^. The International Council on Harmonisation Q1b report^4^ gives the procedures adopted for stress testing and photostability of new drug substances and products. It is therein recommended that photostability studies are conducted on the API both in its pure chemical form, and in its final pharmaceutical formulation. These considerations significantly contribute to the quality and safety of pharmaceuticals over their lifetime from production to patient, including technical production, storage, manipulation, administration and within the patient body^1–5^. Such photostability studies are also mandatory considering both the large number of photosensitive drugs, and the effects of light on their potency, efficacy, and adverse biological effects^1–3,6–10^. Drugs’ photodegradation also raised serious concerns about the impact their presence or the presence of their photoproducts may have in the environment^11,12^.

Despite the extended documentation in the drug photodegradation field, there were only a few interesting discussions in relation to the procedures, the metrics and the overall quality of the existing photodegradation data^1–3,9–12^. For instance, the ICH Q1b report^4^ does not specify how the kinetic data collected on drugs’ photodegradation should be treated in order to measure the reactions’ parameters. Also, the literature does not provide consensus on whether the data treatment varies with regard to the photomechanism undergone by the drug or the experimental conditions employed. In this regard, there is no indication whether the type of light (mono- or polychromatic) requires any variation in the photokinetic treatment procedures or mathematical formulae. Finally, there are no databases for photodegradation metrics of drugs.

The quantification of drugs’ photodegradation basically relies on kinetic studies. In general, the most often used approach, is the classical methodology that was designed for (thermal) chemical kinetics. Within this remit, some studies have introduced some modifications on the treatment (e.g., using sometime numerical integration methods, or introducing power series in the rate-law^2,3^). One can observe that even when modifications are introduced, the end formulae turn out to correspond to the general mathematical formulation of the classical chemical kinetics used for thermal reactions (mostly zeroth- or first-order kinetic model equations). It is however important to underline here that, despite their ubiquity, these formulae do not derive from closed-form integration of the rate-laws describing drugs’ photodegradation. Such a situation raises a legitimate question on whether photodegradation of drugs must obey the classical kinetic type of reaction orders and whether its data must be treated according to that methodology.

It has previously been shown^13^ that the reaction kinetics of the primary photoprocess whose photoproduct is transparent to the monochromatic irradiation light, $AB{(1\Phi )}_{{\varepsilon }_{B}=0}$, obeys the Φ-order kinetics. Mathematically, the timely evolution of the concentration, $c=f(t)$*,* is defined by a logarithmic function that carries an exponential term in its argument. The Φ-order kinetics defines a new reaction behaviour that is different from the ones undergone by pure thermal reactions.

It was also acknowledged that when the photoproduct of the unimolecular reaction absorbs the excitation light, i.e. $AB{(1\Phi )}_{{\varepsilon }_{B}\ne 0}$ systems as Nifedipine and Dacarbazine^14,15^, the solution of the rate-law is not possibly derived analytically. This meant that the true kinetic order of this reaction is, thus far, not accessible. However, a robust semi-empirical approach has facilitated an explicit $\left(c=f(t)\right)$ expression of the Φ-order kinetics type^14,15^ for the integrated rate-law for the $AB{(1\Phi )}_{{\varepsilon }_{B}\ne 0}$ reaction subjected to monochromatic light. Some of the relevant results facilitated by this approach were (i) the devising of a rationale and methodology based on simple integrated rate-laws, (ii) the quantification of the photoprotection of drugs that is induced by absorption competitors, (iii) the proof of self-photostabilisation of drugs by the exclusively increase of the drug’s initial concentration, (iv) the evidenced increase of the photoreaction rate with decreasing initial concentration of the reactant, and (v) the establishment of a simple actinometric procedure to convert drugs obeying $AB{(1\Phi )}_{{\varepsilon }_{B}\ne 0}$ photomechanism into efficient and reliable actinometers for monochromatic light.

Our work on monochromatic light irradiation, was also extended to photoreversible drugs such as Montelukast, Sunitinib and oxyresveratrol^16–19^, and multi-consecutive photodegradation reactions such that of Riboflavin^20^. For these cases, semi-empirical methods allowed to derive $c=f(t)$ algebraic formulae as explicit integrated rate-law equations obeying Φ-order kinetics.

Such an effort could be seen as a contribution to both establish a reliable methodology for photodegradation studies, and to harmonise the area of quantifying drugs’ photodegradation with specific and well-defined metrics.

Within this objective, there is still a gap in the knowledge that needs to be addressed. Notwithstanding the progress that has been achieved on modelling drugs’ phtodegradation under monochromatic light, there is a lack of $c=f(t)$ kinetic equations for drugs exposed to polychromatic light. Most often, the kinetic behaviour under polychromatic light is assumed to obey the classical 0th- or 1st-orders of thermal reactions^1–3^. It is obvious that if the latter assumption does not apply to monochromatic light (i.e. the Φ-order kinetics template applies, as discussed above^13–20^), it is reasonable to conjecture that it would, most likely, not apply when polychromatic light irradiation is considered.

The rate equation of a photoreaction subjected to a polychromatic irradiation involves an integral over the domain of the irradiation light, over a range Δλ, reaching the reactive medium (where *i* species may be present). The integrand includes the absorbed light and the quantum yield, both quantities corresponding to the individual wavelengths belonging to Δλ ($\int {\Phi }^{\lambda }\left({A}_{j}^{\lambda }/\sum {A}_{i}^{\lambda }\right)\left({E}_{0}^{\lambda }/V\right)\left(1-1{0}^{-\sum {A}_{i}^{\lambda }}\right)d\lambda$), Δλ^21–24^, with ${A}_{j}^{\lambda }$, the absorbance of species *j* for which the rate is set out, and ${E}_{0}^{\lambda }$, the spectral irradiance of the lamp incident light (photon count) at wavelength $\lambda$. It is, however, interesting to notice that the above integrand was variably formulated, for instance, by replacing the term ${E}_{0}^{\lambda }$ by the product ($\lambda {E}_{beam}^{\lambda }$) where ${E}_{beam}^{\lambda }$ represented the energy of the light beam^25^, or by (${E}_{0}^{\lambda }/\lambda$)^26^, or by (${E}_{0}^{\lambda }/\sum {E}_{0}^{\lambda }$)^27^, with, in this case, the spectral irradiance being expressed in W cm^−2^ nm^−1^, or by (${E}_{0}^{\lambda }/t$)^28^, where *t* is the integration time for the measurement of the lamp’s emission spectrum. Recently, a rate equation, claimed to be a general chemical rate expression, for any photoreaction irradiated by a polychromatic light source, omitted the ratio $\left({A}_{j}^{\lambda }/\sum {A}_{i}^{\lambda }\right)$ from the integral given above, and considered the exponential factor to be equal to $1{0}^{-{A}_{i}^{\lambda }}$^29^. Another form of the rate equation was based on only initial and final absorbances of the medium^30^. Despite their differences, these differential rate equations are, unfortunately, not analytically solvable. Usually, the kinetic trace is calculated by numerical integration^21–29^. As a consequence, there are no analytically established simple algebraic equations, of the form $c=f(t)$, to map out the kinetic traces and the behaviour of reactions under polychromatic light. As a matter of fact, in this area, the proposal of algebraic equations with the aim to describing photokinetics under polychromatic light, necessarily means that such equations are approximations. It is, however, always more convenient to handle algebraic equations in kinetics^31^, so that the reaction features such as the order of the reaction, the rate-constant and/or the initial velocity, can be easily evaluated (such kinetic criteria are not all offered by numerical integration). The condition set out for the use of such algebraic equations is to predict, interpret and describe well the measured kinetic traces at hand^31^. This strategy has been adopted by methods based on initial-rate equations for the determination of the quantum yield (supposed wavelength-invariant) of photoreactions under polychromatic light^25,26^, or by proposing a *proxy* integrated rate-law of the reaction investigated^27,32^ (in all cases, the algebraic equations were not analytically derived from the differential rate equations as set above). It is also interesting to notice that despite the differences in rate laws and/or descriptive algebraic equations, the methods proposed in these studies have individually shown excellent replication of the experimental kinetic data of the reactions studied.

In the present study, the investigation of polychromatic light induced kinetics is considered for the photoreaction $AB{(1\Phi )}_{{\varepsilon }_{B}\ne 0}$. The algebraic equations derived for this reaction are applied to the photodegradations of two drugs Dacarbazine and Nifedipine. In ethanol, the respective photodegradations of these drugs produce a single photoproduct^14,15^. Since, the photoproducts spectra overlap those of their respective reactants, their photoreactions belong to the $AB{(1\Phi )}_{{\varepsilon }_{B}\ne 0}$ family.

The anti-cancer drug Dacarbazine (DBZ), is used for the treatment of metastatic malignant melanoma, Hodgin’s disease and soft tissue sarcoma^33–38^. Nifedipine is an anti-hypertensive drug, a dihydropyridine that belongs to one of the three groups of calcium-channel blockers, i.e. benzothiazepines, dihydropyridines and phenylalkylamines^1–3,39–42^.

Both drugs are known to undergo fast photodegradations. Their kinetics have usually been fitted to 0th- or 1st-order models, irrespective of whether the light employed was mono- or polychromatic^1–3,9,43–48^. In aqueous solutions, their photodegradations produce species responsible of phototoxic effects^1–3,9,46^. In this respect, developing robust kinetic strategies for both monochromatic and polychromatic light irradiations, will allow improving assessment and control of photodegradation.

## Experimentals

### Chemicals

Dacarbazine (DBZ), 5-(3,3-dimethyl-1-triazeno)imidazole-4-carboxamide, and Nifedipine (NIF), 3,5-dimethyl 2,6-dimethyl-(2nitrophenyl)-1,4-dihydropyridine-3,5-dicarboxylate, and ethanol were purchased from Sigma-Aldrich.

### Analytical solutions

Stock solutions of the drugs in ethanol, were prepared by weighing the solid. They were later used to prepare fresh diluted analytical solutions for experiments performed at various polychromatic irradiation conditions. Volumetric flasks and spectrophotometric cuvettes were protected from ambient light by aluminium foil wrapping.

The fresh solutions of the drugs had each the same concentration for actinometric experiments. They were exposed to the polychromatic light of a series of different intensities. The kinetic traces were collected and subsequently fitted with the appropriate equations.

### Polychromatic irradiation

**Figure 1:**
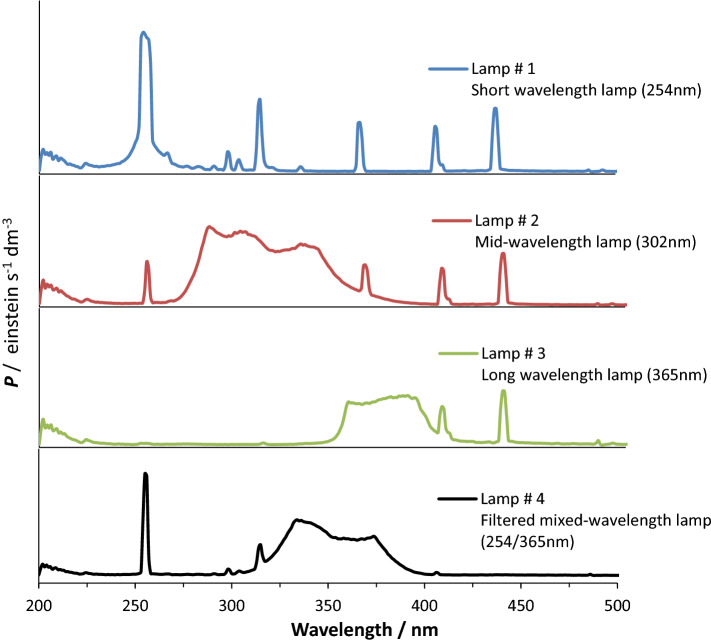
Light profiles of the different light sources used in this study. The profiles were measured on an Avantes spectroradiometer.

Four different lamps were used for the irradiation of the studied samples. Their light profiles (Fig. 1) indicate emissions at different wavelength intervals, as indicated by the manufacturer, Fisher Scientific. These are Lamp #1, the 254-nm short-wavelength lamp model G6T/SW, Lamp #2, the 302-nm mid-wavelength range lamp model G6T5E, Lamp #3, the 365-nm long-wavelength lamp model F6T5, and Lamp #4, the 254/365-nm mixed-wavelength lamp model G6T5HC. These light sources had a 6 W power.

For the purpose of the experiment, each of the above lamps was housed in designed handle, which is placed on the top of the irradiation cabinet (UPV C10-E6 mini UV viewing contrast contro). The inside of the cabinet is totally shielded from external light. This instrument was supplied by Fisher Scientific.

The studied solution, held in the 1-cm cuvette reactor and continuously stirred, was placed inside the irradiation cabinet, maintained at a given position under the lamp where the surface of the solution was perpendicular to the incident light beam.

Notice that if the lamps were labelled by a particular wavelength (254, 302, 365 and 254/365, Fig. 1), which might suggest that their emissions are either of a monochromatic character or focused on a particular wavelength, these lamps effectively deliver polychromatic lights that span several, relatively wide, regions of the electromagnetic spectrum.

The reduction of the light intensity, required for actinometric investigation, was achieved by placing one or more copper grid-mesh tiles above the reactor (as light intensity attenuating filters).

The sides of the reactor-cell (apart from the top 1-cm^2^ section) were covered with aluminum foil, as to allow the lamp light to impinge on the sample from the top (collimated light irradiation supposed not to undergo scattering within the sample). The volume of the sample irradiated in the reactor was 2 mL.

For each experiment, the incident radiation profile of the selected lamp was measured using the spectroradiometer. Reaction media were removed from the cabinet at set irradiation time intervals**,** and analysed by either or both spectrophotometery and HPLC.

The lamps’ profiles were obtained using an Avantes spectroradiometer model Avaspec/UL2048CL/EVO/50, using a UA-grating (200–1100 nm). The light beam reached the spectroradiometer though a slit-25 with a 400 μm optical fiber after correction by an in-line cosine corrector model FC/UVIR/1/BX and CC-UV/Vis. The collected profile intensities have the dimension mW/cm^2^/nm which has been converted into einstein/dm^3^/s as the unit of the photon flux per irradiated volume (${P}_{0}$) required by photokinetics (Fig. 1).

Measurements involving wavelength have been performed at 1 nm steps.

### HPLC analyses

A Perkin Elmer HPLC instrument using a reversed-phase Waters symmetry (C18 150 mm × 3.9 mm, 5μ) column, equipped with a Perkin Elmer Series 200 pump, UV/Vis detector, vacuum degasser and a Perkin Elmer type Chromatography Interface 600 series Link linked to a computer system, was used.

The mobile phases were obtained by automatically mixing two solvents. The mobile phase for NIF, was 55% methanol and 45% deionised water, that of DBZ, was 5% acetonitrile and 95% deionised water. A flow rate of 1 ml/min and an injection loop of 20 μl were employed for both drugs. The UV-detector wavelength was set to 326 nm for NIF and 330 nm for DBZ.

**Figure 2:**
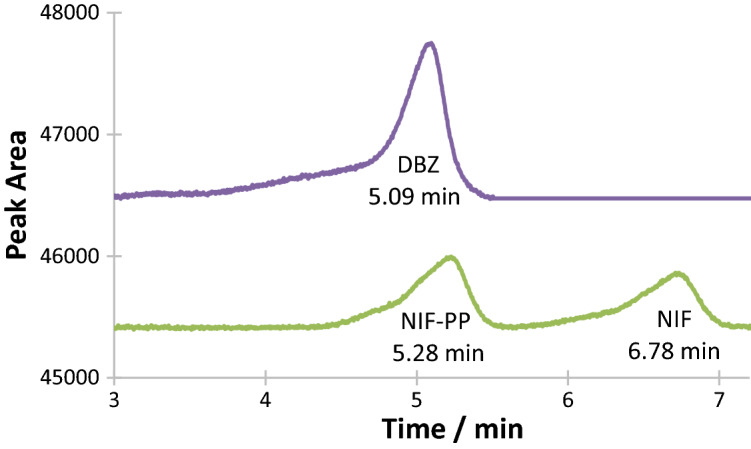
HPLC-Chromatograms of Dacarbazine and Nifedipine measured at absorption wavelengths of 330 and 326 nm, respectively. Note that the photoproduct, DBZ-PP, does not absorb at 330 nm.

The retention times of initial molecule and photoproduct were 6.86 and 5.28 min for NIF, and 5.09 min for DBZ (Fig. 2). The respective calibration graphs of the mother compounds (Table 1) were built using peak areas (for HPLC), selected single wavelengths (spectrophotometry), or sum of absorbances (spectrophotometry).

**Table 1:** Calibration features of the drugs.

Drug	Calibration graph	Linearity range × 10^–5^ /M	Correlation coefficient, *r*^2^
***Spectrophotometry (absorbance at a single wavelength)***			
DBZ	4996($\pm$ 2.31%) × C + 0.0007	1.50–10.74	0.999
NIF	22,545($\pm$ 0.88%) × C − 0.0628	1.50–13.20	0.999
***HPLC (peak area)***			
DBZ	4($\pm$ 1.16%) × 10^9^ × C − 18,040	1.72–27.50	0.999
NIF	2($\pm$ 1.96%) × 10^9^ × C − 10,810	0.90–50.0	0.999
***Spectrophotometry (sum of absorbances)***			
DBZ	1,150,804.8($\pm$ 0.88%) × C + 0.16	1.50–10.74	0.999
DBZ-PP*	345,940($\pm$ 0.89%) × C + 0.041		0.999
NIF	1,920,969.95($\pm$ 1.16%)x C − 4.79	1.50–13.20	0.999
NIF-PP*	1,937,923.35($\pm$ 1.50%)x C − 4.68		0.999

*PP is the photoproduct of the drug.

All experiments were conducted, at least, in triplicates.

## Mathematical background and order of reaction under polychromatic light irradiation

The primary photoreaction, generally labelled $AB(1\Phi ),$ involves the phototransformation of a reagent (**A**) into a product (**B**), i.e., **A** —*h*ν → **B**. Despite its simplicity, it is important to notice that, from a reaction photokinetics viewpoint, the primary photoprocess can give rise to two cases in relation to whether or not the photoproduct absorbs the incident light. For the former, the photoproduct is transparent to the light beam used for irradiation of the sample, i.e. the absorptivity of **B** is zero over the range of wavelengths used for irradiation (${\varepsilon }_{B}=0$). It has been labelled $AB{(1\Phi )}_{{\varepsilon }_{B}=0}$. In the latter kinetic case, **B** absorbs the incident light at the same time as **A**. This photochemical system has the label $AB{(1\Phi )}_{{\varepsilon }_{B}\ne 0}$.

The distinction between these two cases is relevant because their differential equations (rate-laws) are different. This means that the solutions of rate-laws, i.e. the integrated rate-laws, are necessary different. This general principle is also evident, for instance, for thermal reactions where the integrated rate-law of zeroth-order reactions is a linear function whereas that of first-order reactions is described by a mono-exponential function. A variation that stems primarily from the differences occurring in their respective rate-laws.

The aforementioned mathematical principle as well as the analogy with thermal reactions, suggest that the photoreactions $AB{(1\Phi )}_{{\varepsilon }_{B}=0}$ and $AB{(1\Phi )}_{{\varepsilon }_{B}\ne 0}$ kinetic orders are expected to be different.

For an $AB{(1\Phi )}_{{\varepsilon }_{B}=0}$ system subjected to a monochromatic light, the closed-form integration of the corresponding rate-law was found to obey a pure Φ-order kinetics^13^. But the rate-law of the second $AB{(1\Phi )}_{{\varepsilon }_{B}\ne 0}$ photosystem under the same type of light is not possibly integrated in a closed-form (there are no available mathematical methods capable to deriving a solution in a close-form for this non-linear differential equation). A semi-empirical approach has however proposed an explicit formula for the integrated rate-law of this $AB{(1\Phi )}_{{\varepsilon }_{B}\ne 0}$ whose formulation is typical of Φ-order kinetics with a condition that the final absorbance at the irradiation wavelength does not exceed 0.6^14^.

The case $AB{(1\Phi )}_{{\varepsilon }_{B}=0}$ is very rare for drugs as the spectra of APIs generally overlap those of their photoproducts. From this perspective, the $AB{(1\Phi )}_{{\varepsilon }_{B}\ne 0}$ is a more common mechanism for drugs (e.g., NIF and DBZ). However, a closed-form integration of the rate-law of a polychromatic-light driven $AB{(1\Phi )}_{{\varepsilon }_{B}\ne 0}$ photosystems has never been proposed in the literature.

### A proposed integrated rate-law for $AB{(1\Phi )}_{{\varepsilon }_{B}\ne 0}$

The proposed rate-law describing $AB{(1\Phi )}_{{\varepsilon }_{B}\ne 0}$ reactions, when polychromatic light is employed for irradiation (Eq. 1), must take into account the wavelength (${\lambda }_{j}$) interval spun by the polychromatic excitation light (${\lambda }_{a}\le {\lambda }_{j}\le {\lambda }_{b}$, i.e. $\Delta \lambda ={\lambda }_{b}-{\lambda }_{a}$). In the case where the total absorbance is high because of a high total absorptivity (> 10^5^–10^6^ M^−1^ cm^−1^, as for NIF and DBZ, Table 1), and a relatively high total incident radiation intensity of the lamp (~ 10^–4^ einstein dm^-3^ s^-1^), an approximation of the rate-law can take the form,

1 $$\frac{{C}_{A}\left(t\right)}{dt}=-\left(\sum_{{\lambda }_{j}={\lambda }_{a}}^{{\lambda }_{b}}{\Phi }_{A\to B}^{{\lambda }_{j}}\times {\varepsilon }_{A}^{{\lambda }_{j}}\times {P}_{0}^{{\lambda }_{j}}\right)\times {l}_{irr}\times {F}^{\Delta \lambda }(t)\times {C}_{A}\left(t\right)$$

where ${C}_{A or B}\left(t\right)$ the concentrations (in *M*) of **A** or **B** at time t (in *s*) and ${l}_{irr}$ (in *cm*) is the optical path length of the collimated irradiation light inside the reactive medium. The summation is carried out over the product of the photochemical quantum yield of the primary photoreaction (${\Phi }_{A\to B}^{{\lambda }_{j}}$, dimensionless), the photon flux per unit volume of the incident light (${P}_{0}^{{\lambda }_{j}}$, expressed in *einstein dm*^−3^* s*^−1^), and the absorption coefficient of **A** (${\varepsilon }_{A}^{{\lambda }_{j}}$, in *mol*^−1^ *dm*^*3*^* cm*^−1^). The latter quantities are given here relative to an individual wavelength, ${\lambda }_{j}$ within the range $\Delta \lambda$.

The photokinetic factor, ${F}^{\Delta \lambda }(t)$ (dimensionless), must take into account the time dependent, multi-wavelength absorptions (${A}_{A or B}^{{\lambda }_{j}}(t)$) of the light by both reagent (**A**) and photoproduct (**B**). The total absorbance, in this case, is given by

2 $${A}_{tot}^{\Delta \lambda }\left(t\right)=\sum_{{{\lambda }_{j}=\lambda }_{a}}^{{\lambda }_{b}}\left({A}_{A}^{{\lambda }_{j}}\left(t\right)+{A}_{B}^{{\lambda }_{j}}\left(t\right)\right)=\sum_{{{\lambda }_{j}=\lambda }_{a}}^{{\lambda }_{b}}{\varepsilon }_{A}^{{\lambda }_{j}}\times {l}_{irr}\times {C}_{A}\left(t\right)+{\varepsilon }_{B}^{{\lambda }_{j}}\times {l}_{irr}\times {C}_{B}\left(t\right)$$

where, ${F}^{\Delta \lambda }(t)$ takes the form

3 $${F}^{\Delta \lambda }(t)=\frac{1-{10}^{-{A}_{tot}^{\Delta \lambda }\left(t\right)}}{{A}_{tot}^{\Delta \lambda }\left(t\right)}$$

The total absorbance, ${A}_{tot}^{\Delta \lambda }\left(t\right)$ in Eq. 2, should in practice have a numerical value exceeding, by far, unity when $\Delta \lambda$ exceeds a few nanometers. Hence, if ${A}_{tot}^{\Delta \lambda }\left(t\right)\gg 1$ then ${10}^{-{A}_{tot}^{\Delta \lambda }\left(t\right)}\ll 1$ and ${F}^{\Delta \lambda }(t)$, considering the mass balance, can be reduced to

4 $${F}^{\Delta \lambda }(t)=\frac{1}{{A}_{tot}^{\Delta \lambda }\left(t\right)}=\frac{{\alpha }_{1}^{\Delta \lambda }}{{C}_{A}\left(t\right)+{\alpha }_{2}^{\Delta \lambda }}$$

with ${\alpha }_{1}^{\Delta \lambda }$ and ${\alpha }_{2}^{\Delta \lambda }$ are both constants (expressed in *M*) for a given $\Delta \lambda$, and defined as

5 $${\alpha }_{1}^{\Delta \lambda }=\frac{1}{{l}_{irr}\times \sum_{{{\lambda }_{j}=\lambda }_{a}}^{{\lambda }_{b}}\left[{\varepsilon }_{A}^{{\lambda }_{j}}-{\varepsilon }_{B}^{{\lambda }_{j}}\right]}$$

6 $${\alpha }_{2}^{\Delta \lambda }={C}_{A}\left(0\right)\times \frac{\sum_{{\lambda }_{j}={\lambda }_{a}}^{{\lambda }_{b}}{\varepsilon }_{B}^{{\lambda }_{j}}}{\sum_{{\lambda }_{j}={\lambda }_{a}}^{{\lambda }_{b}}\left[{\varepsilon }_{A}^{{\lambda }_{j}}-{\varepsilon }_{B}^{{\lambda }_{j}}\right]}$$

By introducing Eq. (4) in Eq. (1), and rearranging the obtained rate-law to separate the variables, we obtain

7 $$\frac{{C}_{A}\left(t\right)+{\alpha }_{2}^{\Delta \lambda }}{{C}_{A}\left(t\right)}{dC}_{A}\left(t\right)=- {\alpha }_{1}^{\Delta \lambda }\times {l}_{irr}\times \left(\sum_{{\lambda }_{j}={\lambda }_{a}}^{{\lambda }_{b}}{\Phi }_{A\to B}^{{\lambda }_{j}}\times {\varepsilon }_{A}^{{\lambda }_{j}}\times {P}_{0}^{{\lambda }_{j}}\right) dt$$

Equation 7 can be solved by closed-form integration to yield the integrated rate-law (Eq. 8).

8 $$\left({C}_{A}\left(t\right)-{C}_{A}\left(0\right)\right)+{\alpha }_{2}^{\Delta \lambda } ln\frac{{C}_{A}\left(t\right)}{{C}_{A}\left(0\right)}=- {\alpha }_{1}^{\Delta \lambda }\times {l}_{irr}\times \left(\sum_{{{\lambda }_{j}=\lambda }_{a}}^{{\lambda }_{b}}{\Phi }_{A\to B}^{{\lambda }_{j}}\times {\varepsilon }_{A}^{{\lambda }_{j}}\times {P}_{0}^{{\lambda }_{j}}\right)t$$

The latter integrated rate-law (Eq. 8) could be presented in a much simpler form,

9 $$\eta \left(t\right)=- {k}_{\eta }\times t$$

The left-hand side term of the integrated rate-law (Eq. 9), combines linear and logarithmic terms has the dimension of a concentration (*M*).

10 $$\eta \left(t\right)=\left({C}_{A}\left(t\right)-{C}_{A}\left(0\right)\right)+{\alpha }_{2}^{\Delta \lambda } ln\frac{{C}_{A}\left(t\right)}{{C}_{A}\left(0\right)}$$

and in its right-hand side term, ${k}_{\eta }$ has the dimension *M s*^−1^, and is expressed as

11 $${k}_{\eta }=\frac{\sum_{{\lambda }_{j}={\lambda }_{a}}^{{\lambda }_{b}}{\Phi }_{A\to B}^{{\lambda }_{j}}\times {\varepsilon }_{A}^{{\lambda }_{j}}\times {P}_{0}^{{\lambda }_{j}}}{\sum_{{\lambda }_{j}={\lambda }_{a}}^{{\lambda }_{b}}\left[{\varepsilon }_{A}^{{\lambda }_{j}}-{\varepsilon }_{B}^{{\lambda }_{j}}\right]}$$

### The kinetic order of the polychromatic-light driven $AB{(1\Phi )}_{{\varepsilon }_{B}\ne 0}$ photoreaction

The simple formulation of the integrated rate-law (Eq. 9), with its coefficients (Eqs.10 and 11), does not compare to any known integrated rate-law proposed, to date, in kinetics. The $\eta \left(t\right)$ expression (Eq. 10) is a mixture of a zeroth-order kinetics (the linear section), and a first-order kinetics (the logarithmic section). Whereas, the ${k}_{\eta }$ constant has a dimension of a zeroth-order reaction. Such a combination of kinetic orders in one formula has never been observed before for a single reaction.

The dimension of ${k}_{\eta }$ can be thought as a strong argument to consider it as a rate-constant for the $AB{(1\Phi )}_{{\varepsilon }_{B}\ne 0}$ photoreaction. In its form, Eq. (11) indicates that the numerical value of ${k}_{\eta }$ should increase with quantum yield and incident light intensity. A specific property of photoreactions that has also been identified for the photoreaction’s rate-constant obeying a pure Φ-order kinetics ($AB{(1\Phi )}_{{\varepsilon }_{B}=0}$ under monochromatic-light irradiation)^13,14^.

Accordingly, Eq. (9) describes the particular kinetic behaviour of $AB{(1\Phi )}_{{\varepsilon }_{B}\ne 0}$ photoreactions, and hence, defines a new reaction order: the η-kinetic order.

As a characterisation of η-order kinetics, let us look at some of its properties.

(i) the applicability of η-order kinetics is limited to the linearity range of the calibration graph built using the variation of ${A}_{tot}^{\Delta \lambda }$. In the case, $\sum_{{\lambda }_{j}={\lambda }_{a}}^{{\lambda }_{b}}{\varepsilon }_{A}^{{\lambda }_{j}}> \sum_{{\lambda }_{j}={\lambda }_{a}}^{{\lambda }_{b}}{\varepsilon }_{B}^{{\lambda }_{j}}$, a calibration graph according to Eq. (12) is sufficient. Otherwise, ($\sum_{{\lambda }_{j}={\lambda }_{a}}^{{\lambda }_{b}}{\varepsilon }_{A}^{{\lambda }_{j}}< \sum_{{{\lambda }_{j}=\lambda }_{a}}^{{\lambda }_{b}}{\varepsilon }_{B}^{{\lambda }_{j}}$) a similar calibration graph to Eq. (12) should be built for the photoproduct alone (*n,* in Eq. (12), being the number of different initial concentrations used for the calibration graph).
  12 $${A}_{A}^{\Delta \lambda ,n}\left(0\right)=\left(\sum_{{\lambda }_{j}={\lambda }_{a}}^{{\lambda }_{b}}{\varepsilon }_{A}^{{\lambda }_{j}}\times {l}_{irr}\right)\times {C}_{A}^{n}\left(0\right)$$
(ii) point (i), generally imposes that the initial concentration of the reactant is relatively low. The Beer-Lambert law, which only applies over the linearity range of the calibration graph, discourages the use of high concentrations of the photoreactant.
(iii) ${k}_{\eta }$ is independent of the initial concentration of reactant **A**
(iv) ${k}_{\eta }$ is dependent on the irradiation wavelength interval
(v) ${k}_{\eta }$ is independent of the optical path length of irradiation (${l}_{irr}$)
(vi) ${k}_{\eta }$ is proportional to ${\Phi }_{A\to B}^{{\lambda }_{j}}$, and ${P}_{0}^{{\lambda }_{j}}$
(vii) The half-life time (${t}_{1/2}$ in *s*), defined by Eq. (13), is not only dependent on the initial concentration (${C}_{A}\left(0\right)$) of the reactant (**A**) but also on all the attributes of the photochemical reaction, including the absorptivities of both reagents (**A** and **B**), and $\Delta \lambda$. At constant ${C}_{A}\left(0\right)$, ${t}_{1/2}$ is expected to decrease with increasing radiant power. ${t}_{1/2}$ is proportional to ${C}_{A}\left(0\right)$.
  13 $${t}_{1/2}=\frac{0.5\times {C}_{A}\left(0\right)+{\alpha }_{2}^{\Delta \lambda } ln2}{{k}_{\eta }}$$
(viii) The initial velocity of the reactant’s reaction (${r}_{0,A}^{\eta }$ in *M s*^−1^), given by Eq. (14), is obtained from Eq. (1). It has similar dependencies on the reaction attributes as ${k}_{\eta },$ e.g., ${r}_{0,A}^{\eta }$ increases with increasing incident light intensity. It is expected to be independent of the initial concentration if the term ($1-{10}^{-{A}_{tot}^{\lambda }\left(0\right)}$) is equal to unity, i.e., when ${A}_{tot}^{\Delta \lambda }\left(0\right)\gg 1$.
  14 $${{r}_{0,A}^{\eta }=\left[\frac{{dC}_{A}\left(t\right)}{dt}\right]}_{t=0}=- {C}_{A}\left(0\right)\times {k}_{\eta }\times {l}_{irr}\times \sum_{{\lambda }_{j}={\lambda }_{a}}^{{\lambda }_{b}}\left[{\varepsilon }_{A}^{{\lambda }_{j}}-{\varepsilon }_{B}^{{\lambda }_{j}}\right]\times {F}^{\Delta \lambda }(0)$$
(ix) Finally, it is important to underline that the $AB{(1\Phi )}_{{\varepsilon }_{B}\ne 0}$ photoreaction undergoes a true change of kinetic order, depending on the type of light employed. $AB{(1\Phi )}_{{\varepsilon }_{B}\ne 0}$ systems obey pure first-order kinetics if they are subjected to isosbestic monochromatic light. They follow a (semi-empirical) Φ-order kinetics when the irradiation light is monochromatic but non-isosbestic. These reactions obey η-order kinetics if the light driving the phototransformation is polychromatic.

## Results and discussion

### The photokinetics of DBZ and NIF under monochromatic light

Photodegradations of both drugs under monochromatic light irradiation, preformed in ethanol solutions, were previous reported^14,15^. It was established therein that each drug underwent a purely unimolecular photoreactions yielding its corresponding, single photoproduct (either NIF-PP or DBZ-PP). These reactions are typical $AB{(1\Phi )}_{{\varepsilon }_{B}\ne 0}$. The electronic absorption spectra of reactant and photoproduct overlapped on a large section of the absorption domain except for the long wavelength UVA-Vis region. The data collected on their respective photodegradations showed that these drugs obeyed Φ-order kinetics.

It was also demonstrated that the quantum yields of their respective phototransformations were wavelength-dependent following sigmoid trends (the highest values of the quantum yield were situated towards the higher wavelengths). The quantum yield values of DBZ and NIF over $\Delta \lambda$ will be used for our investigation of the polychromatic light irradiation of these drugs.

Extra experiments have confirmed the predictions of the mathematical Φ-order model^14,15^: on one hand, a well quantified self-photostabilisation was induced by increasing initial reactant concentration, and on the other hand, a significant improvement of drug photoprotection was quantified in the presence of absorption competitors.

### Spectral features of DBZ and NIF under polychromatic light

**Figure 3:**
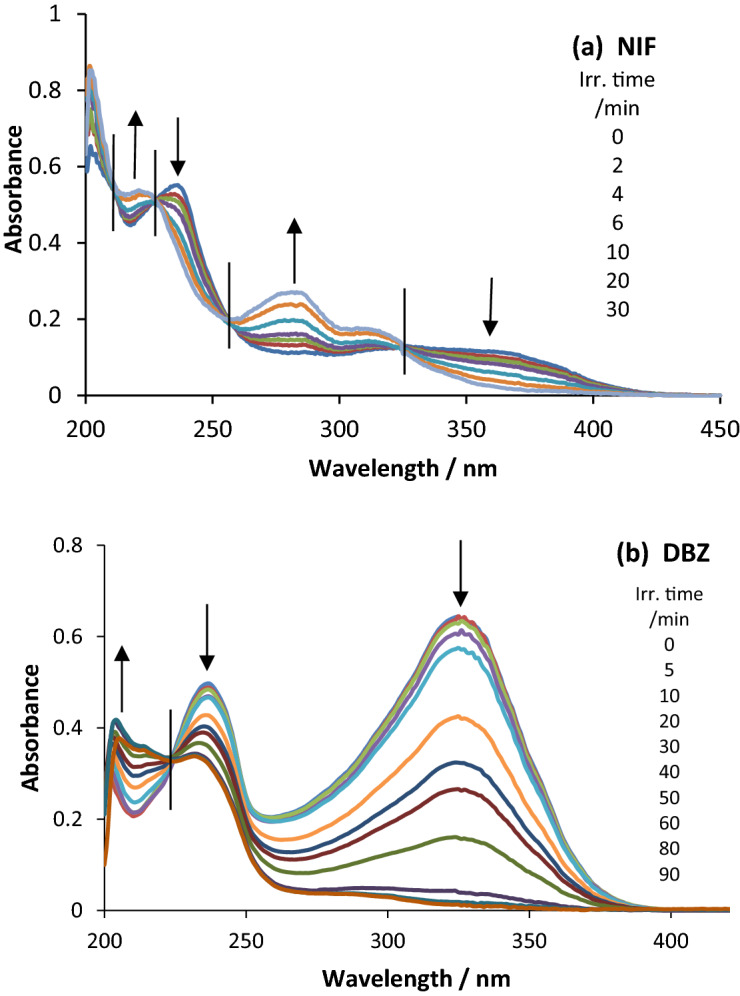
Evolution of the absorption spectra of (**a**) NIF (2.67 × 10^–5^ M) and (**b**) DBZ (5.41 × 10^–5^ M) in ethanol, when subjected to increasing irradiation time intervals. The polychromatic light (200–400 nm) was delivered by a mixed-wavelength lamp (254/365 nm, Fig. 1). Arrows indicate direction of absorbance change; vertical lines cross the spectra at the isosbestic points.

Qualitatively, the changes observed on the electronic spectra the drugs during polychromatic light exposure (Fig. 3) were very similar to those induced by monochromatic irradiation^14,15^. Incidentally, such a similarity meant that it is not possible to know, from the kinetic traces, which type of irradiation was used to drive the photoreactions (implying that the characterisation of a monochromatic light can only be achieved by instrumentation, e.g., monochromators).

The mechanism in play for both drugs does not change due to the nature the irradiation light (poly- or monochromatic), as confirmed by the occurrence of a single photoproduct, for each drug, identified by HLPC over reaction times.

The progress of the photoreaction is evidenced by a reduction in absorbances of the lowest-energy π → π* electronic absorption bands, and an increase of most of the remaining transitions. The clearly identified isosbestic points indicate smooth drugs’ photodegradation with no measurable presence of by-products (Fig. 3). Little evolution of the spectra is recorded at the end of the reactions indicating that the reactant-drug (either NIF or DBZ) has been fully depleted.

The last electronic spectrum recorded at the end of the reaction ($t=\infty$), corresponds therefore, to that of the photoproduct. The measured total absorptions coefficients of **A** and **B** ($\sum_{{\lambda }_{a}}^{{\lambda }_{b}}{\varepsilon }_{A}^{{\lambda }_{j}}$ and $\sum_{{\lambda }_{a}}^{{\lambda }_{b}}{\varepsilon }_{B}^{{\lambda }_{j}}$) are 1,150,028.96 and 344,785.15 M^−1^ cm^−1^, and 1,920,219.56 and 1,936,173.24 M^−1^ cm^−1^ for the pairs DBZ/DBZ-PP and NIF/NIF-PP, respectively. Notice that the former values for the spectra of DBZ show that $\sum_{{\lambda }_{a}}^{{\lambda }_{b}}{\varepsilon }_{B}^{{\lambda }_{j}}$ (i.e., of DBZ-PP) is much smaller than that of DBZ. This is not the case for NIF whose total absorption coefficient is practically the same as that of its photoproduct (less than 0.9% difference, which was also later confirmed from their calibration graphs, Table 1). This means that in both cases a calibration graph of the reactant is sufficient for our study.

### Kinetics of the drugs under polychromatic light

**Figure 4:**
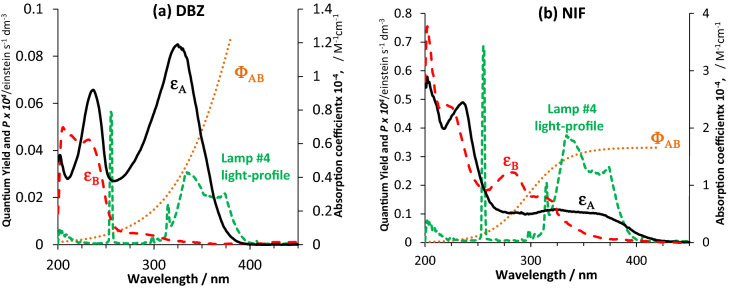
Absorption coefficients of initial drug (ε_**A**_) and its photoproduct (ε_**B**_) for (**a**) DBZ and (**b**) NIF, the mixed (254/365 nm) wavelength light-profile of the irradiation lamp, and the sigmoid patterns of the photochemical reactions’ quantum yields^14,15^.

The irradiation of the ethanolic solutions of both drugs has been performed by a mixed-wavelength lamp (Lamp #4) that has emissions at both 254 nm and over the 310–400 nm range (Figs.1 and 4). The span of the light supplied by this lamp, overlaps the regions of electronic absorption of both drugs.

The increasing quantum yields with wavelength (Fig. 4) indicate that both drugs are more photodegradable with UVA rather than with UVB. Overlapping this data with Lamp #4 profile and absorption spectra of the species, makes it evident that most photoreactivity will be due to the reactant molecule transforming under UVA/VIS light (the UVB light of Lamp #4 occurring mainly at 254 nm, would only have a marginal contribution). Such an analysis of the data is mandatory to explain and understand the kinetic behaviour of drugs under polychromatic light (a further analysis is presented in “Methodology to devising new drug-actinometers for $AB{(1\Phi )}_{{\varepsilon }_{B}\ne 0}$ systems under polychromatic light” section).

Timely irradiations of the samples with polychromatic light causes a smooth decrease of the long-wavelength electronic bands (Fig. 3). HPLC analysis confirmed that the photoproducts are photochemically stable, at least, for longer times than the experiments’ durations.

The kinetic traces, recorded at maximum absorbance wavelengths, suggest a fast photodegradation in the early stages (< 15 min), followed by a more curved pattern at later times, in full agreement with the predictions of the η-order kinetics model (Eqs.9, and 10).

**Figure 5:**
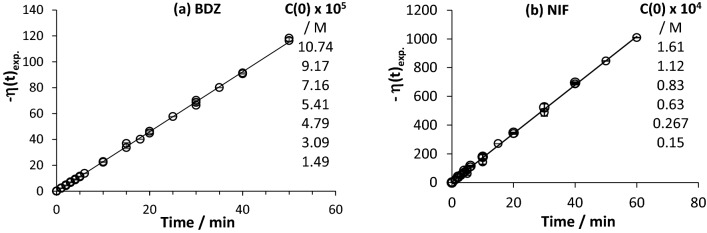
Linear η-order correlations (according to Eq. 9) for the experimental photokinetic traces of (**a**) DBZ and (**b**) NIF, measured at various initial concentrations and exposed to Lamp #4. The error bars indicate an RSD less than 2%.

The experimental data from these traces, of both drugs, were well described by the η-order kinetic model (Eq. 9), with η(t) values obtained for different concentrations, evolve according to linear relationships with photoreaction time (Fig. 5).

Qualitatively, the experimental overall rate-constants (${k}_{\eta }$), corresponding to the gradient of η-order kinetics, $\eta \left(t\right)=-{k}_{\eta } t$, and performed in the same experimental conditions for both drugs, are higher for NIF than for DBZ (${k}_{\eta ,NIF}^{Lamp\#4}>{k}_{\eta ,DBZ}^{Lamp\#4}$, Fig. 5). NIF, is hence, much more photodegradable than DBZ under irradiation by the same mixed-wavelength Lamp #4. A finding that corroborates the relative photoreactivity of these drugs under monochromatic light, according to the ranking scale that was established based on each drug’s range of its β-factor over its absorption region^15–20^. This scale was thought to set the foundation for a database on photoactive drugs.

For NIF and DBZ the β-factor values ($2121\le {\beta }_{DBZ}\le 4013$ and $8117\le {\beta }_{NIF}\le 9776$ M^−1^ s) attested of a much higher reactivity of NIF compared to DBZ (in that classification, NIF and DBZ belong to group II and III, respectively)^19,20^.

The overall rate-constants (${k}_{\eta }$) could then play, for polychromatic light, the same role played by β-factor for monochromatic light. Such a scale, starting here with the ${k}_{\eta }$ values of these two drugs under Lamp #4, would be very useful to inform about drugs photoreactivity with regard to light type (the usefulness of such a scale is also relevant to actinometry, as described further in “Methodology to devising new drug-actinometers for $AB{(1\Phi )}_{{\varepsilon }_{B}\ne 0}$ systems under polychromatic light” section).

### The effect of initial concentration of DBZ and NIF on photodegradation

In previous studies^14,20,49,50^, the effect of initial concentration on Φ-order kinetics was considered for strictly monochromatic-light irradiation. It was experimentally shown that the rate-constant of the photoreaction decreases with increasing initial concentration for the direct reaction $AB{(1\Phi )}_{{\varepsilon }_{B}\ne 0}$ as in the cases of NIF^14^ and Nisoldipine^49^, and predicted for the photorevesrible $AB(2\Phi )$^50^ and multi-consecutive $A{B}_{4}(4\Phi )$^20^ reactions as indicated by the corresponding formulae of the respective rate-constants. Incidentally, the overall rate-constant of the pure primary photoreaction, $AB{(1\Phi )}_{{\varepsilon }_{B}=0}$^13^, does not depend on initial concentration of the reactant.

These findings strongly suggest that the initial concentration of the reactant might have a significant impact on the kinetic rates of photoreactions. Thus far however, there are no systematic proofs in the literature on how ${C}_{A}(0)$ affects the overall reactivity when the irradiating beam is a polychromatic light.

**Figure 6:**
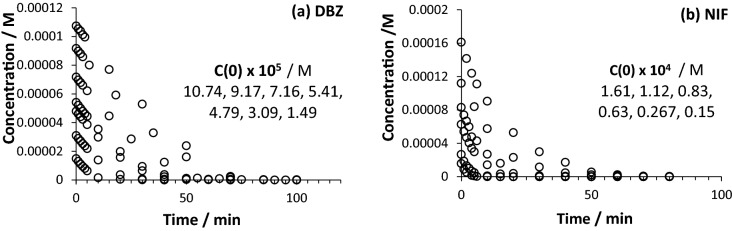
Photokinetic traces of (**a**) DBZ and (**b**) NIF under polychromatic light using the mixed-wavelength lamp #4 (254/365 nm). The experimental data (circle) were determined by HPLC.

For the present study, the effect of increasing initial concentrations of DBZ and NIF has been investigated in the respective ranges 1.5–10.5 × 10^–5^ M, and 1.5–16.1 × 10^–5^ M (Fig. 6). The selected concentrations span the full linearity ranges of the drugs’ calibration graphs.

The η-order kinetic formulae (Eqs. 9–11,14) allowed the determination of the individual η-order overall rate-constants (${k}_{\eta }$) and initial velocities (${r}_{0}^{\eta }$) for each kinetic trace recorded for NIF and DBZ. An excellent correlation (less than 5% error) was found between these experimental values and the ones independently calculated using Eqs.11 and 14. Such an agreement of experimental and calculated values for ${k}_{\eta }$ or ${r}_{0}^{\eta }$, is a solid confirmation of the validity of the mathematical η-order kinetics model, proposed here for $AB{(1\Phi )}_{{\varepsilon }_{B}\ne 0}$ phototransformations, since the data used for the calculated values and those of the experimental ones belong to separate studies involving different radiation conditions^14,15^.

**Figure 7:**
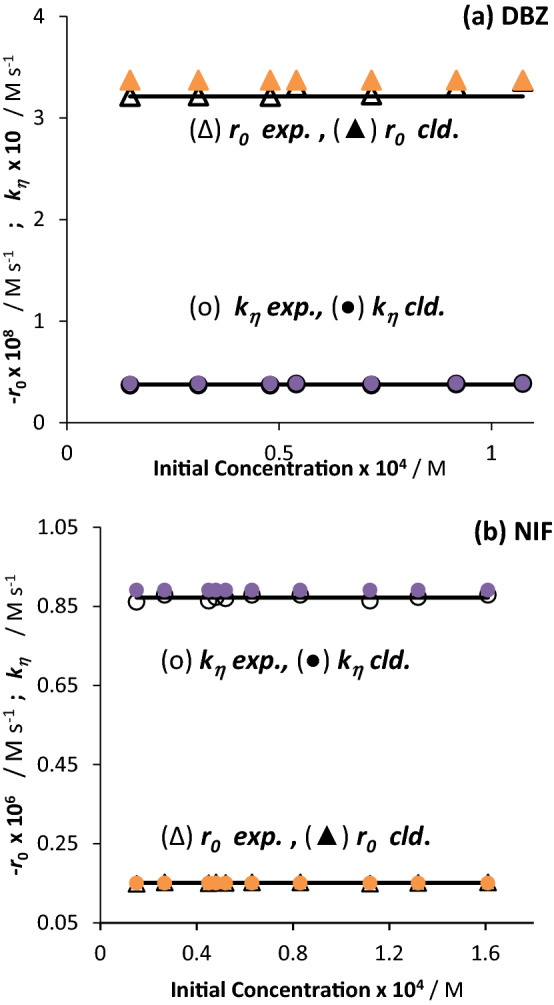
Invariance of ${k}_{\eta ,\mathrm{exp}.}$ and ${r}_{0,exp}^{\eta }$ for (**a**) DBZ and (**b**) NIF with different initial concentrations $({C}_{A}(0))$. The solid symbols correspond to the calculated values of ${k}_{\eta ,cld.}$ and ${r}_{0,cld.}^{\eta }$. The ${k}_{\eta ,\mathrm{exp}.}$ and ${r}_{0,exp}^{\eta }$ have, respectively, $\pm$ 1σ of 0.007 and 0.06 for DBZ, and 0.0012 and 0.007 for NIF.

Representing of ${k}_{\eta }$ or ${r}_{0}^{\eta }$ against initial reactant concentration proved the invariance of both these quantities with initial concentration of the drugs (Fig. 7, Eqs.11, and 14); in an obvious contradiction to what has been predicted and observed for monochromatic light irradiation. Hence, with regard to the variation of initial concentration, an $AB{(1\Phi )}_{{\varepsilon }_{B}\ne 0}$ reaction adopts different behaviours depending on the (mono- or polychromatic) type of the light used.

Nonetheless, this conclusion is valid as long as the Beer-Lambert law is also valid, i.e. ${C}_{A}(0)$ must fall within the linearity range of the drug’s calibration graph.

### Methodology to devising new drug-actinometers for $AB{(1\Phi )}_{{\varepsilon }_{B}\ne 0}$ systems under polychromatic light

The potential of DBZ and NIF for polychromatic light actinometry is tested by subjecting freshly prepared solutions of given concentration (2.3 × 10^–5^ M and 2.69 × 10^–5^ M, respectively, in ethanol) to varying total intensities of the polychromatic light (${P}_{0,tot}^{\Delta \lambda }=\sum_{{\lambda }_{a}}^{{\lambda }_{b}}{P}_{0}^{{\lambda }_{j}}$) emitted by the mixed-wavelength range lamp (Lamp #4). Total (over $\Delta \lambda$) instead of individual (at each $\lambda$) incident light intensity of the lamp is used here, to conform with the procedure generally adopted in actinometry. The photokinetic experimental traces for each irradiation (“*q*” at ${P}_{0,tot}^{\Delta \lambda ,q}$) were constructed based on HPLC measurements of the reactant concentrations. These traces were analysed according to η-order kinetics (Eq. 9) and the experimental ${k}_{\eta ,q}$ values corresponding to each individual total incident light intensity, ${P}_{0,tot}^{\Delta \lambda ,q}$, were determined.

**Figure 8:**
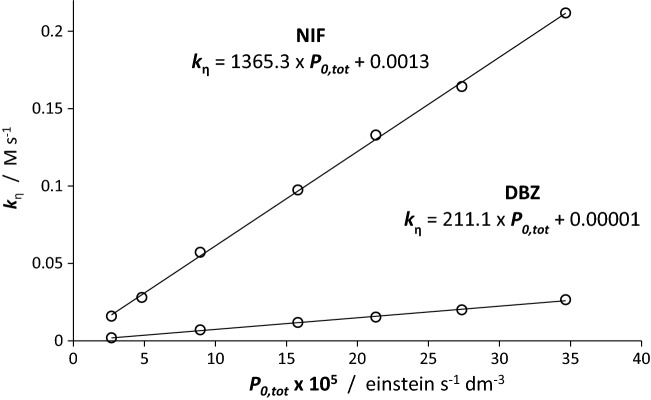
Linear correlations between experimental values of the overall rate-constant (${k}_{\eta }$) and the total radiant power (${P}_{0,tot}^{\Delta \lambda }$) of Lamp #4 for DBZ and NIF in ethanolic solutions (2.3 and 2.69 × 10^–5^ M, respectively) at room temperature.

A perfectly linear relationships linked variations of the overall rate-constant and the total light intensity for both investigated drugs with relatively high (*r* > 0.99) correlation coefficients of the lines, with intercepts close to zero (Fig. 8). The experimentally constructed graphs in Fig. 8 conform well to the principle predicting an increasing photoreactivity with incident light intensity as expected for ${k}_{\eta }$ (see above point (vi)). Nonetheless, such a correlation might come as a surprise, because such a linear trend is not obvious from the formula of ${k}_{\eta }$ (Eq. 11, which explicitly depends on individual ${P}_{0}^{{\lambda }_{j}}$). In fact, since Eq. (11) predicts that a specific value of ${k}_{\eta ,q}$ will correspond to a given irradiation with total ${P}_{0,tot}^{\Delta \lambda ,q}$ , Eq. (11) can be re-written for that purpose, as linear relation of ${k}_{\eta ,q}$ and ${P}_{0,tot}^{\Delta \lambda ,q}$ for any Lamp (Eq. 15). The ${\beta }_{\eta }$ factor is a constant when ${P}_{0,tot}^{\Delta \lambda ,q}$ varies for given experimental conditions and reactive system (i.e., the ratio $\sum_{{\lambda }_{j}={\lambda }_{a}}^{{\lambda }_{b}}\left({\Phi }_{A\to B}^{{\lambda }_{j}} {\varepsilon }_{A}^{{\lambda }_{j}} {P}_{0}^{{\lambda }_{j}}\right)/\sum_{{{\lambda }_{j}=\lambda }_{a}}^{{\lambda }_{b}}{P}_{0}^{{\lambda }_{j}}$ in Eq. (15) is invariant with change of the incident light intensity of the lamp).

15 $${k}_{\eta ,q}=\frac{\sum_{{{\lambda }_{j}=\lambda }_{a}}^{{\lambda }_{b}}{\Phi }_{A\to B}^{{\lambda }_{j}}\times {\varepsilon }_{A}^{{\lambda }_{j}}\times {P}_{0}^{{\lambda }_{j}}}{{P}_{0,tot}^{\Delta \lambda ,q} \sum_{{{\lambda }_{j}=\lambda }_{a}}^{{\lambda }_{b}}\left[{\varepsilon }_{A}^{{\lambda }_{j}}-{\varepsilon }_{B}^{{\lambda }_{j}}\right]} {P}_{0,tot}^{\Delta \lambda ,q} = {\beta }_{\eta ,Drug}^{Lamp \#4} {P}_{0,tot}^{\Delta \lambda ,q}$$

According to Eq. (15), the gradients of the lines in Fig. 8, represent the coefficients ${\beta }_{\eta ,DBZ}^{Lamp \#4}$ and ${\beta }_{\eta ,NIF}^{Lamp \#4}$ of the drugs for the employed Lamp #4. The fact that ${\beta }_{\eta ,NIF}^{Lamp \#4}$ is almost 6.5-fold higher than ${\beta }_{\eta ,DBZ}^{Lamp \#4}$ (respectively, 1365 and 211 M^-1^ s, Fig. 7), clearly indicates that NIF photodegradation under Lamp #4 is faster than that of DBZ. Also, owing to the invariance of ${k}_{\eta }$ with initial concentration (“The effect of initial concentration of DBZ and NIF on photodegradation” section), the same behaviour and these values of ${\beta }_{\eta }$ factors should be expected irrespective of the initial concentration of the drugs.

A strong evidence for reliability of the η-order kinetics model comes from the calculated coefficients ${\beta }_{\eta ,drug}^{Lamp \#4}$ using Eq. (15) and the experimental attributes including individual wavelength quantum yield values determined in previous investigations^14,15^. For both investigated drugs, less than 5% variation was recorded between these calculated ${\beta }_{\eta ,cld., drug}^{Lamp \#4}$ and the experimental (${\beta }_{\eta ,exp., drug}^{Lamp \#4}$) ones.

The latter finding is paramount, because it proves unnecessary the experimental actinometric measurements for $AB{(1\Phi )}_{{\varepsilon }_{B}\ne 0}$ systems under polychromatic irradiation. Indeed, it implies that whenever individual-wavelength attributes are available for actinometers, actinometric reaction and lamp, the actinometric behaviour under polychromatic light of the given lamp, can surely and easily be calculated from Eq. (15), with no need to actually performing the corresponding experiments. A considerable advantage in the field, that underlines another benefit of a detailed wavelength study of the photoreaction under monochromatic light. Incidentally, a very early prediction,^14^ has foreseen the determinant involvement of such monochromatic-light data in the modelling of polychromatic kinetics.

Figure 8 also confirms that the photokinetic behaviour of actinometers is intimately impacted by both their intrinsic photochemical and spectrophotometric features, even when they are irradiated by the same lamp (Eq. 15).

Also obvious from Fig. 8, that individual measurements of the rate-constant ${k}_{\eta }$ have to be considered specific to the experiment at hand, because as such they cannot allow drawing general conclusions about the relative reactivity of the species (only the $\beta$ factors can allow this type of conclusions, as above seen with ${\beta }_{\eta ,NIF}^{Lamp \#4}>{\beta }_{\eta ,DBZ}^{Lamp \#4}$). For instance, an individual ${k}_{\eta }$ value of DBZ can well be higher than that of NIF if the light intensity impinging on the former is higher than that shone on the latter (e.g., ${k}_{\eta ,DBZ}=0.0265 M{s}^{-1}$ (${\mathrm{P}}_{0,\mathrm{tot}}^{\Delta\uplambda ,\mathrm{DBZ}}=34.7 1{0}^{-5} M {s}^{-1}$) and ${k}_{\eta ,NIF}=0.0158 M{s}^{-1}$ (${\mathrm{P}}_{0,\mathrm{tot}}^{\Delta\uplambda ,\mathrm{NIF}}=2.7 1{0}^{-5} M {s}^{-1}$); however, always ${k}_{\eta ,NIF}>{k}_{\eta ,DBZ}$ if the light intensity is the same).

Now, in terms of using these drugs as actinometers, the following procedure can be adopted. This actinometric method is developed here for DBZ and NIF but can be extended to any photosystem of the $AB{(1\Phi )}_{{\varepsilon }_{B}\ne 0}$ type. The purpose of this method is the determination of the unknown total light intensity of a Lamp X. It can be summarised in two major (A and B) steps:

-(A) the actinometer-drug is calibrated with Lamp X. The drug is calibrated using various known intensities of Lamp X. The ${\beta }_{\eta ,drug}^{Lamp X}$ is determined in a similar way presented above (Fig. 8). ${\beta }_{\eta ,drug}^{Lamp X}$ is archived for actinometric purposes in future experiments. For the instance of lamp #4, the ${\beta }_{\eta ,drug}^{Lamp \#4}$ factors, determined in the present study of the drugs, can be used without a need for re-measurements, as proposed in this point (A), if the conditions of the experiment are the same. This, however, is not easy to achieve between different laboratories, and so recalibration is most often necessary (using step B).

-(B) the selected actinometer-drug is used to determine an unknown light intensity of Lamp X, ${P}_{0,tot}^{\Delta \lambda ,unk}$. The actinometer sample is irradiated by Lamp X, as for the investigated reaction of interest.

The detailed procedure can be performed according to the following chart.

(act-1) prepare a fresh solution of either DBZ or NIF in ethanol at a concentration between 1.5–10.5x10^-5^ M and 1.5–16.1x10^-5^ M, respectively.

(act-2) irradiate the sample with Lamp X at a known light-intensity value, ${P}_{0,tot,i}^{\Delta \lambda }$. Record the kinetic trace until no more variation is detected on the absorption spectrum, and on the HPLC chromatograms.

(act-3) use the previous data to calculate the corresponding value for ${\eta }_{i}\left(t\right)$

(act-4) draw ${\eta }_{i}\left(t\right)=- {k}_{\eta ,i}\times t$, for the data at hand (the graph should be a straight line).

(act-5) determine the value of ${k}_{{\eta }_{i}}$ as the gradient of the previous line (act-4).

(act-6) repeat steps (act-2) to (act-5) using $$n$$ ($$n>3)$$ different light intensities of the same lamp, irradiating the sample at the same selected concentration.

(act-7) draw ${k}_{\eta ,i}={\beta }_{\eta ,drug}^{Lamp X}\times {P}_{0,tot,i}^{\Delta \lambda }$ and determine the value of the gradient ${\beta }_{\eta ,drug}^{Lamp X}$ as a reference value for this actinometer.

(act-8) subject a fresh sample of the actinometer, as the ones used previously, to irradiation from Lamp X, with currently unknown intensity ${P}_{0,tot}^{\Delta \lambda ,unk}$, used for the actual experiment.

(act-9) determine the value of ${k}_{\eta ,unk.}$ as described in (act-2) to (act-5).

(act-10) calculate the unknown total light intensity of Lamp X, ${P}_{0,tot}^{\Delta \lambda ,unk}$, for the actual experiment, using Eq. (16).

16 $${P}_{0,tot}^{\Delta \lambda ,unk}=\frac{{k}_{\eta ,unk.}}{{\beta }_{\eta ,drug}^{Lamp X}}$$

These examples of DBZ and NIF actinometers are recommended to amend the Q1b ICH document^4^ as alternatives to the quinine hydrochloride actinometer. The usefulness of the latter for reliable actinometry in pharmaceutics has been questioned by a number of studies.^51–53^.

### Are $AB{(1\Phi )}_{{\varepsilon }_{B}\ne 0}$ photosystems universal actinometers?

Actinometers are usually supposed to be universal. Universality here means that the actinometric data obtained for a given actinometer, on one particular lamp, is useful to determine the total light intensity of any other lamp. This is why the actinometers proposed in the literature, even though they have, most often, been calibrated against a single lamp, they are supposed to be useful to directly determine the unknown intensity of any other lamp. This is the case for the ICH recommended actinometer, the quinine hydrochloride^4^, and the much more popular ferrioxalate actinometer. Nonetheless, the above definition of “actinometer universality”, has never been validated by strong experimental evidence.

To test the hypothesis of universality of actinometers, let us consider the photoconversion of NIF under irradiation by different lamps.

For this study we selected 4 different lamps (Fig. 1) whose emissions overlap only small sections of the wavelength range 200–400 nm, namely, (#1) short-wavelength lamp 254 nm, (#2) mid-wavelength lamp 302 nm, (#3) long-wavelength lamp 365 nm and (#4) mixed-wavelength lamp with a filter for visible light 254/365 nm. Each lamp was used to perform a full actinometric study as described in the previous section.

**Figure 9:**
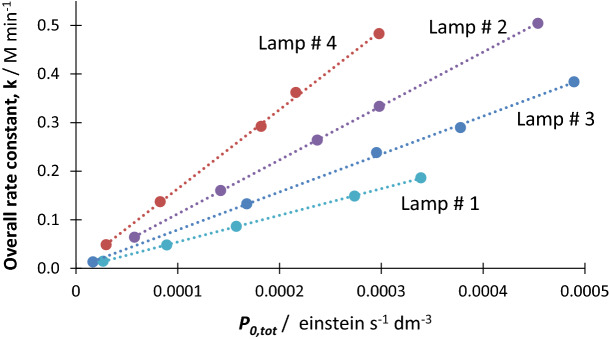
Linear relationships obtained for NIF (2.69 × 10^–5^ M in ethanol) between the overall rate constants, ${k}_{\eta }$, measured for various lamps intensities and the corresponding total radiant power, ${P}_{0,tot}^{\Delta \lambda }$, for the four lamps (Lamp #1–Lamp #4, Fig. 1).

Identical total radiant powers $({P}_{0,tot}^{\Delta \lambda , Lamp \#i})$ from the different lamps were also provided for the irradiation of NIF samples. The ${\beta }_{\eta ,NIF}^{Lamp \#i}$ factors were determined for each lamp separately as shown in Fig. 9 (and Table 2).

**Table 2:** Correlation features of NIF overall rate-constant, ${k}_{\eta }$, with the total radiant power, ${P}_{0,tot}^{\Delta\lambda }$, for the four lamps used in this study (Fig. 1).

Lamp # *i*	Line equation* ${k}_{\eta }^{Lamp \#i}$ =${\beta }_{\eta ,NIF}^{Lamp \#i}\hspace{0.17em}\times \hspace{0.17em}{P}_{0,tot}^{\Delta \lambda , Lamp \#i}$ + intercept	*r*^2^
1	548.26 ${P}_{0,tot}^{\Delta \lambda , Lamp \#1}$ − 0.4 10^–3^	0.99
2	1109.8 ${P}_{0,tot}^{\Delta \lambda , Lamp \#2}$ + 1.2 10^–3^	0.99
4	1365.3 ${P}_{0,tot}^{\Delta \lambda , Lamp \#3}$ + 1.3 10^–3^	0.99
3	779.26 ${P}_{0,tot}^{\Delta \lambda , Lamp \#4}$+ 1.5 10^–3^	0.99

*The RSD of the gradients do not exceed 3%

It is evident that the different lamps have different effects on the photokinetics of NIF. The highest rate of photodegradation was observed for Lamp #4 (${\beta }_{\eta ,NIF}^{Lamp \#4}=1365.3$) and the lowest for lamp #1 (${\beta }_{\eta ,NIF}^{Lamp \#1}=548.3$). These can be explained by the emission range of the lamp relative to the absorption spectrum and quantum yield of NIF (Figs.1 and 4). Accordingly, the main emission region of the short wavelength lamp (~ 254 nm) corresponding to the lowest section of NIF quantum yield sigmoid (200–275 nm, Fig. 4), explains well the lowest ${\beta }_{\eta }$ factor value recorded for this Lamp #1. The remaining light-sources’ profiles overlap the highest quantum yield region. Lamp #4 profile (325–400 nm) covers almost fully the higher quantum yield section (300–400 nm, Fig. 4), compared to Lamps #2 and #3. Lamp #4 records the highest ${\beta }_{\eta }$ value. However, the last two lamps (#2 and #3) are not that evident to rank within the set. If the profile of Lamp #2 overlaps a 100 nm of the quantum yield sigmoid, Lamp #3, overlaps *ca*. 60 nm of the sigmoid plateau region (350–400 nm, Figs.1 and 4) corresponding to the highest quantum yield values.

Since such a qualitative analysis cannot help to rank Lamps #2 and 3, then let us use the $\eta$-order model. The sum of products, the $\sum (\Phi \varepsilon P)$ term, in the rate-constant formula (Eq. 15), easily explains the difference in ${k}_{\eta }$ values that are recorded for either changing species that are irradiated with the same lamp (Fig. 8), or changing lamp profiles for the same species (Fig. 9). It is, in both cases, highly unlikely that the sums ($\sum (\Phi \varepsilon P)$) be equal. For instance, the qualitative interpretation offered above for the ranking of Lamps #2 and #4 ${k}_{\eta }$ values, are predicted by Eq. (15).

For Lamps #2 and 3, Eq. (15) can give an interpretation of the difference in their experimental ${k}_{\eta }$ values. Indeed, let us first note that the lamp profile #3 coincides with a spectral region where practically only the reactant absorbs, whereas, Lamp #2 emission spans both reactant and photoproduct absorption regions, specially over the 275–350 nm section. The denominator of Eq. (15) increases with the difference between the total absorbances, $\sum \left({\varepsilon }_{A}-{\varepsilon }_{B}\right)$, of reactant and photoproduct. Therefore, ${k}_{\eta }$ and hence ${\beta }_{\eta ,NIF}^{Lamp \#i}$ decreases as $\sum \left({\varepsilon }_{A}-{\varepsilon }_{B}\right)$ tends to its maximum value, $\sum {\varepsilon }_{A}$. Accordingly, Eq. (11) predicts that the overall rate-constant values for Lamp #3 (where almost only the reactant absorbs, $\sum {\varepsilon }_{B}\cong 0$) should most likely be lower than that of Lamp #2. Therefore, we reconstitute the correct experimental order of ${\beta }_{\eta ,NIF}^{Lamp \#i}$ values (Fig. 9).

The above discussion confirms the validity of $\eta$-order equations but does not tell whether the NIF-actinometer is universal. For this let us consider the scenario where NIF-actinometery was developed on a particular lamp (let it be Lamp #4 with ${\beta }_{\eta ,NIF}^{Lamp \#4}$ its constant, Table 2) and the test lamp could be any of the set (Lamp #1 to #4).

Obviously, if the test lamp is #4 then there are no issues and the unknown intensity could be worked out as described in the above procedure (act-10).

More interestingly is to consider the case when the test lamp is different from #4 (let it be Lamp #2). The question here is whether the unknown intensity of Lamp #2 can be precisely determined by exposing our NIF-actinometer sample to this lamp, and determining its unknown light intensity using an earlier calibration performed on Lamp #4.

Let us consider that the overall rate-constant recorded experimentally for NIF with the test lamp (#2) is, for instance, ${k}_{\eta ,\#2}=0.332 M {s}^{-1}$. The corresponding light intensity of this ${k}_{\eta ,\#2}$ value on Lamp #4 calibration is ${P}_{0,tot,cld.}^{\Delta \lambda , Lamp \#4}=2.42 1{0}^{-4} M {s}^{-1}$. The latter value ($2.42 1{0}^{-4} {s}^{-1}$) is supposedly expressing the total light intensity received by NIF from Lamp #2 (whose kinetics is characterised by ${k}_{\eta ,\#2}=0.332 M {s}^{-1}$). This is not correct since the latter value (${k}_{\eta ,\#2}$), on the specific calibration of Lamp #2 (Fig. 9, Table 2), corresponds to a total intensity of light, ${P}_{0,tot}^{\Delta \lambda , Lamp \#2}=2.98 1{0}^{-4} M {s}^{-1}$. This clearly means that there is a difference of *ca.*19% between the true value of the total light intensity inducing the reaction (Lamp #2, ${k}_{\eta ,\#2}$ and ${P}_{0,tot}^{\Delta \lambda , Lamp \#2}$), and that worked out from the Lamp #4-based actinometry (Lamp #4, ${k}_{\eta ,\#2}$ and ${P}_{0,tot}^{\Delta \lambda , Lamp \#4}$). A Similar situation arises if one of the other lamps (#1 or #3) is used instead of Lamp #2.

Therefore, one is forced to accept that it is simply not possible to work out the correct ${P}_{0,tot}^{\Delta \lambda , Lamp \#i}$ ($i\ne j$) from that of an actinometer that was calibrated with Lamp #j (for our example *i* = #4 and *j* = #1, #2 or #3).

Accordingly, NIF is not a universal actinometer. It is necessary to use it as an actinometer only for the lamp it was calibrated with.

A finding that makes up an experimental proof stating that polychromatic-light actinometers cannot be universal. Each actinometer/lamp pair must be calibrated individually, i.e., for a given actinometer, and hence, there are as many calibration methods required as there are light sources. This has been proven here for $AB{(1\Phi )}_{{\varepsilon }_{B}\ne 0}$ systems, but in principle, should be true for all actinometers. Therefore, the working principle should, indeed, become “actinometry and actinometers are lamp-specific”.

Since the conditions of irradiation vary from lab to lab (light source, size of the irradiated sample area and sample volume), then it would be recommended to develop the actinometric method on site using the procedure (act-1 and act-10).

## Concluding remarks and perspectives

Our experimental results for NIF and DBZ actinometries confirm that can be proposed as reliable actinometers for the UVA range of polychromatic light sources, and alternatives to the ICH Q1b actinometer. NIF and/or DBZ actinometry will provide reliable and more accurate measurements for pharmaceutical photostability studies. The low concentrations needed for such actinometry considerably reduces the cost of these methods. They also open an avenue to recruiting more $AB{(1\Phi )}_{{\varepsilon }_{B}\ne 0}$ drug-actinometers to allow widening the spectral range covered.

The η-order kinetics is proven to be an effective way to describe $AB{(1\Phi )}_{{\varepsilon }_{B}\ne 0}$ reactions when exposed to polychromatic light. In this context, the classical kinetic (0th-, 1st- and 2nd-) orders become, de facto, invalid for this type of reactions and undoubtedly lead to incorrect results and conclusions.

A new perspective in photokinetics and actinometer emerges from the present study, that is, rate-laws and integrated rate-laws are both mechanism and light condition specific which leads actinometry to being both lamp profile and actinometer, specific.
